# The psychometric properties of the person-centered therapeutic relationship in physiotherapy scale

**DOI:** 10.1371/journal.pone.0241010

**Published:** 2020-11-06

**Authors:** Óscar Rodríguez-Nogueira, Jaume Morera Balaguer, Abel Nogueira López, Juan Roldán Merino, José-Martín Botella-Rico, Sonia Del Río-Medina, Antonio R. Moreno Poyato

**Affiliations:** 1 Department of Nursing and Physiotherapy, SALBIS Research Group, Campus de Ponferrada, Universidad de León, Ponferrada, León, Spain; 2 Physical Therapy Department, CEU Universities, Universidad Cardenal Herrera-CEU, Elche, Alicante; 3 European University of the Atlantic, Santander, Spain; 4 International Ibero-American University, Campeche, Mexico; 5 Department of Sport, International University of Cuanza, Cuito, Angola; 6 Campus Docent, Sant Joan de Déu-Fundació Privada, School of Nursing, University of Barcelona, and Researcher, Research Group GIES (Grupo de investigación en Enfermerıía, Educación y Sociedad), Barcelona, Spain; 7 Research Group GEIMAC (Consolidated Group 2017–1681: Group of Studies of Invarianza of the Instruments of Measurement and Analysis of Change in the Social and Health Areas), Barcelona, Spain; 8 Escola d´Infermeria Departament d'Infermeria de Salut Pública, Salut Mental i MaternoInfantil Facultat de Medicina i Ciències de la Salut, Campus de Bellvitge, Universitat de Barcelona, Pavelló de Govern, Barcelona, Spain; Murcia University, Spain, SPAIN

## Abstract

**Objective:**

To determine the psychometric properties of the Person-Centered Therapeutic Relationship in Physiotherapy Scale (PCTR-PT) in order to find the most appropriate fit for the tool.

**Methods:**

Patients who had received treatment at the physiotherapy service of nine hospitals in Spain were invited to complete the 31 items of the PCTR-PT scale. To select the most appropriate items of the PCTR-PT, an exploratory factorial analysis (EFA) was performed using the maximum likelihood and oblique rotation (promin) methods. Factor validity, goodness-of-fit and psychometric properties were analyzed by confirmatory factor analysis (CFA). Convergent (CFA) and discriminant validity were calculated. Internal consistency was verified using the Cronbach's alpha coefficient. The intraclass correlation coefficient (ICC) was used to examine temporal stability.

**Results:**

366 patients over 18 years old who had received, at least, 15 physiotherapy treatment sessions completed the questionnaire. The results of the exploratory factor analysis revealed a tool with 15 items in four factors [Relational Bond (N items = 4); Individualized Partnership (N items = 4); Professional Empowerment (N items = 3) and Therapeutic Communication (N items = 4)], explaining 78.4% of the variance of the total variables of this tool. The confirmatory factor analysis further confirmed the four-structure model. Reliability of the tool was approved by Cronbach's alpha in all four dimensions, as all were above .70, ranging from .84 (Individualized Partnership) to .91 (Professional Empowerment). = 0.94. Test-retest was performed with two-week intervals, indicating an appropriate stability for the scale (ICC = 0.900).

**Conclusion:**

The Person-Centered Therapeutic Relationship in Physiotherapy Scale (PCTR-PT) is a useful, valid and applicable instrument to evaluate the person-centered therapeutic relationship during physiotherapy interventions. It would be interesting to investigate the predictive capacity (sensitivity and specificity) of the PCTR-PT scale.

## Introduction

Person-centered care is recognized by numerous disciplines as being a standard of quality in clinical practice [1], as well as a goal in itself [2]. Its implementation is considered a priority goal for improving healthcare in the 21st century [3]. Thus, according to the World Health Organization (WHO) [4], the comprehensive needs of individuals and communities, and not just diseases, must be at the center of healthcare systems and therefore professionals should empower people to play a more active role in their own health.

In the year 2000, Mead and Bower [5] conducted a literature review to construct a conceptual framework on person-centered care that was made up of five dimensions: the biopsychosocial perspective, the patient as a person, the sharing of power and responsibility, the therapeutic alliance, and the professional as a person. These authors emphasized that person-centered care ensures higher quality standards in health care, and that its relevant elements include the professional's ability to understand the unique needs of each person and to establish a healthy interpersonal relationship [5]. Subsequently, in 2012, along the same lines, Morgan and Yoder [6] proposed a definition of the concept of person-centered care as a "holistic (bio-psycho-social-spiritual) approach to respectful and individualized care, which makes it possible to negotiate care and offer options through a therapeutic relationship in which the person is empowered to participate in health decisions as he or she wishes". In general, the conceptual framework of person-centered care has been widely described and discussed in the international literature [5–7]. However, the implementation process has not been as well studied [8–11], nor are there measurement tools that directly reflect person-centered participation [12].

Concretely, in the field of physiotherapy, there is still a lack of understanding regarding the meaning and components of the person-centered care concept [13]. A recent systematic review of the construct, with the aim of providing a theoretical framework for developing such care in the field of physiotherapy, concluded that “Patient centeredness in physiotherapy entails the characteristics of offering an individualized treatment, continuous communication (verbal and non-verbal), education during all aspects of treatment, working with patient-defined goals in a treatment in which the patient is supported and empowered with a physiotherapist having social skills, being confident and showing specific knowledge” [13]. These results thus consider the educational aspect throughout the treatment process [13] and once again confirm that, in order to develop person-centered care in physiotherapy, it is necessary to establish adequate communication and a therapeutic relationship between the professional and the person receiving care [7, 13–17]. Moreover, on a multidisciplinary level, the published literature highlights that person-centered care is based on the importance of relational aspects, the individualization of care, empowerment and the sharing of roles and responsibilities through the therapeutic alliance [5–7], this construct comprises three components: 1) the link between the patient and the therapist, 2) agreement on the tasks to be improved, and 3) agreement on the treatment goals [18].

A recent systematic review reported the existence of 11 scales [12] for the measurement of person-centered care. However, after a content analysis, the authors concluded that most tools were not truly focused on direct and proactive patient participation [12], an aspect, once again related to the process of implementation of care, such as therapeutic alliance, engagement and the patients' own experience [8]. In addition, to assess therapeutic interpersonal relationships in clinical practice, the empirical literature has described several measurement instruments such as the Working Alliance Inventory (WAI) [19], the Scale to Assess the Therapeutic Relationship (STAR) [20], the Helping Alliance Questionnaire (HAQ) [21] and the California Psychotherapy Alliance Scale (CALPAS) [22]. These instruments, however, do not incorporate aspects of focused care that are more directed towards the holistic and individualized care of people [6].

Clearly, despite the growing recognition of the value of the therapeutic relationship in the field of physiotherapy [23–25] and the importance of the same for the establishment of person-centered care, there is limited research to guide physiotherapists towards improving this aspect during clinical practice [26]. In addition, its evaluation is difficult due to the lack of specific or appropriate instruments to measure specific associated characteristics during physiotherapy procedures [12, 23, 26–29].

To overcome this situation, a preliminary version of the Person-Centered Therapeutic Relationship in Physiotherapy Scale (PCTR-PT) was designed via a mixed methods study comprising several phases. In phase one, the items of the scale were generated. First, a review and analysis of the literature was performed based on the following constructs: person-centered care and person-centered therapeutic relationship, subsequently, the main items were extracted to create a question guide to explore barriers and facilitators for the establishment of a person-centered relationship in physiotherapy services. Thereafter, a qualitative focus group study was conducted involving 21 physiotherapists [25] and 31 patients of physiotherapy services [29]. With the results obtained, a conceptual framework comprising seven domains and 28 subdomains was built with 215 items. In the second phase, the items were selected using a three-round modified Delphi survey process with nine experts in the field. The experts were selected based on the following criteria: 1) Health professionals interested in the patient-centered therapeutic relationship; 2) With knowledge and experience on this subject; 3) Who had demonstrated their capacity to theorize about the chosen subject, via research projects, theses, articles, communications, etc. during the previous three years. In this phase, the experts were asked to express their degree of agreement regarding clarity, coherence, and relevance for each of the items (1–4; 1 = strongly disagree, 4 = strongly agree). Across the three rounds, we applied the same quantitative selection criteria for each item: 1) a mean score of ≥ 3.25 for degree of agreement, and 2) a rating of 3 or higher for degree of agreement among ≥ 70% of the participants in the Delphi survey. Finally, a questionnaire was designed, based on 31 items, which was used for the subsequent phase. Lastly, the third phase consisted of a cognitive pre-test to assess comprehension, language clarity and content suitability. Two rounds of cognitive interviews were performed with 55 participants from two hospitals within the Spanish public health system and four private physiotherapy centers. The participants in this phase were patients with similar characteristics to those to whom the definitive tool was to be applied. As a result of this phase, four elements were removed and four others added, whereas 16 were reformulated. The final tool comprised 31 items divided into seven domains. The response format was based on a 5-point Likert frequency scale. The response options ranged from “strongly agree” to “strongly disagree. This procedure and the study carried out contributed to the content validity of the PCTR-PT [30]. The aim of the present study was to determine the psychometric properties of the (PCTR-PT) with the ultimate aim of finding the most appropriate fit of the tool.

## Methods

### The (PCTR-PT) scale

The preliminary version of the “Person-Centered Therapeutic Relationship in Physiotherapy (PCTR-PT) scale contains 31 items divided into seven domains: Personal characteristics of the professional (5 items); Communication capacities of the professional (7 items); Professional aspects (4); Relational aspects (6); Personalized therapy (2); Partnership (4); Environment (3). The response format is based on a 5-point Likert frequency scale. Response options range from “strongly agree” (1) to “strongly disagree” (5), with an intermediate option (3): “Neither agree nor disagree”. The preliminary version of the “Person-Centered Therapeutic Relationship in Physiotherapy (PCTR-PT) is show in S1 File.

### Study design and participants

The participants in this phase were patients meeting the following selection criteria: 1) over 18 years old; 2) who had received, at least, 15 physiotherapy treatment sessions; 3) without any cognitive impairments and comprehension difficulties. The participants were recruited from five hospitals within the Spanish public health system (Madrid, A Coruña, Valencia and Alicante) and four private physiotherapy centers (Orense, Alicante, Almería). The physiotherapists from the centers where the respective patients were receiving care were in charge of selecting and inviting the study participants. These health professionals participated voluntarily in the study and were previously informed of the research aims and the inclusion criteria for the researchers during a designated meeting.

During a seven-month period, a sample of consecutive eligible people was identified from the patient register and recruited by the physical therapist attending each patient, who assessed the eligibility criteria, informed each patient of the objectives of the study and the specific implications of their participation, and asked them if they wanted to participate in the study. To stimulate the response rate, we used the hand-delivery technique, which has shown the highest response rates [31]. Once patients were accepted, they were given an envelope with the questionnaire, including explanatory information on the study and its implications, encouraging patients to participate in the research, together with an informed consent form, and a questionnaire for the collection of socio-demographic and clinical data.

A system was established for the collection of anonymous questionnaires that could not be opened by staff at the center, with two urns: one in which they deposited their informed consent, and another in which they deposited an envelope with the socio-demographic questionnaire and the preliminary questionnaire. In addition, reminders were issued. To this end, a call was made to the head of the physiotherapy service of each center, asking him/her to encourage the physiotherapists participating in the study to remind their patients to hand in the questionnaire if they had not already done so. Three verbal reminders were given, at two weeks, and at two and three months.

The study protocol was approved by the Research Ethical Committee of Universidad Cardenal Herrera-CEU, Hospital Universitario 12 de Octubre of Madrid, Hospital General Universitario of Valencia, Complejo Hospitalario Universitario of A Coruña, Hospital General Universitario of Elche, and Hospital Universitario Vinalopó of Elche. This study was performed in accordance with the ethical standards as laid down in the Declaration of Helsinki.

### Statistical analysis

Descriptive statistical analyzes were conducted to assess and conceptualize the demographic and clinical characteristics of the individuals in the analyzed sample. To perform the analysis, the software used was SPSS 25 (an IBM Company, Chicago, IL).

#### Construct validity

In order to determine the factorial structure of the scale, the number of subscales or dimensions and the total of items, an exploratory factorial analysis of the instrument was carried out. For this purpose, the maximum likelihood method was used as the extraction method, together with the oblique (promin) rotation method [32], to attempt to maximize the simplicity of the factors. All items with a factor weight below .40 were removed from the final scale.

Factorial validity, goodness of fit and psychometric properties of PCTR-PT were analyzed by confirmatory factor analysis (CFA) using also the Maximum Likelihood method, which allows the robust calculation of factor structure in terms of fit; from which the values of the Kaiser-Meyer-Olkin index (KMO) and Bartlett's sphericity (X2) were obtained.

The quality of the global fit of the factorial model was calculated according to the following indexes and their respective values, as described by Marôco [33] and Hu & Bentler [34]: the normalized Chi-square, defined as the ratio of the Chi-square value to the number of degrees of freedom (χ2/df), root mean square error of approximation (RMSEA), root mean square residual (RMR), Tucker-Lewis index (TLI), comparative fit index (CFI), goodness-of-fit index (GFI), and the standardized root mean squared residue (SRMR).

Values equal to or less than .05 are considered excellent for RMSEA, RMR, and SRMR; while those less than .08 are acceptable. The X2/df ratio must be < to 3 to establish a correct model, and TLI, CFI and GFI values above .90 or .95 are interpreted as a good fit for the data.

Convergent and discriminant validity was also analyzed. As for the study of the former, the calculation of the Mean Extracted Variance (AVE) was chosen, for which a value greater than .50 indicates adequate convergent validity [35, 36]. However, to confirm the discriminant validity, this was carried out by verifying that the correlations between the constructs are lower than the square root of the mean extracted variance [34, 37].

#### Reliability: Internal consistency

The internal consistency was verified by calculating the Cronbach's alpha coefficient for both the full scale and for each of the subscales, with values of this coefficient equal to or greater than .70 [38, 39]. In turn, to ensure internal consistency, the calculation of the composite reliability of the construction was carried out, where the values greater than or equal to .70, reflect a good consistency [35, 40].

AMOS statistical software (v. 25, SPSS, An IBM Company, Chicago, IL) was used to perform these tests.

#### Temporal stability or test-retest

The temporal stability of the instrument was examined with a subsample of n = 36 selected from the total sample. The questionnaire was re-administered two to three weeks after the first administration, using the intraclass correlation coefficient (ICC), considering .70 values as indicators of acceptable reliability, .80 as good and finally, values above .90 are considered as indicators of excellent reliability [38, 41]

AMOS statistical software (v. 25, SPSS, An IBM Company, Chicago, IL) was used to perform the analyses.

## Results

During a seven-month period, 422 potential participants were identified. Of these, 378 agreed to participate. Finally, 366 completed the questionnaire (87%). The demographics and clinical characteristics of the participants are shown in Table 1. Of the respondents, 59.8% were women, 68% had a mid-level education or higher, 61.8% lived with a partner, 74.3% came from a public or subsidized environment, and the average number of sessions was 31.5 The health problems were: traumatology or rheumatology (78.5%), neurology (4.4%), lymphedema (3.3%), respiratory (1.5%), cardiology (1.5%), sequelae of cancer (0.6%), amputations (0.6%), and other pathologies (hearing loss, aortic aneurysm, vertigo, breast prosthesis encapsulation) (1.4%). In the case of 7.9% of patients, they failed to reflect on the survey which was the health problem for which they sought treatment.

**Table 1:** Socio-demographic and clinical characteristics of the study subjects (n = 366).

SOCIO-DEMOGRAPHIC AND CLINICAL CHARACTERISTICS OF STUDY SUBJECTS) (n = 366)	n	%
Gender		
Male	147	40.2
Female	219	59.8
Mean age in years (SD)	51.7 (SD: 14.56)	
Age		
Between 18 and 40	56	15.3
Between 40 and 60	179	48.9
Over 60	116	31.7
DK/NA	15	4.1
Level of studies		
No education or primary	122	33.3
Middle Studies	127	34.7
University studies	110	30.1
DK/NA	7	1.9
Civil status		
Married/ with a partner	226	61.8
Single	66	18
Separated, divorced or widowed	63	17.2
DK/NA	11	3
Work occupation		
Works	203	55.5
Housewife	33	9
Unemployed	38	10.4
Retired	53	14.5
Student	10	2.7
DK/NA	29	7.9
Diagnosis		
Traumatology/Rheumatology	287	78.5
Neurology	16	4.4
Lymphedema	12	3.3
Cardiology	6	1.5
Respiratory	6	1.5
Sequalae of cancer	2	0.6
Amputation	2	0.6
Others	5	1.4
DK/NA	30	8.2
Treatment center		
Hospital	247	67.5
Public	23	6.3
Others	96	26.2
Scope		
Public	215	58.7
Private	94	25.7
State subsidized private	57	15.6
Mean number of total treatment sessions (SD)	31.5 (SD = 27.51)	
DK/NA	66	

DK/NA: don’t know/no answer

### Factorial structure

Prior to the CFA analysis, multivariate normality values were examined, determining that the values were at the expected level. The structure of the PCTR-PT model was tested using a Structural Equation Model with the Maximum Likelihood method under the scope of CFA. As a result of the analysis, it was determined that the PCTR-PT model fit index values were at an acceptable level. Cronbach's alpha (α) internal consistency coefficient and corrected item total correlation coefficients were examined in reliability analysis of PCTR-PT.

The results of the factor analysis showed that the data set was adequate for EFA [(KMO) coefficient = .876, Bartlett Sphericity Test (χ2) = χ2 = 3894,728, p < .000)]. After the analysis, the factor load values of the items, were examined, together with the overlap and the screen plot. In line with this information, 16 items showed a factor load value less than .40.

Several items were eliminated since many had loads on multiple factors, (convergent validity), and in none of these did the load reach the minimum value of .30 [35, 38]. Likewise, the Cronbach's alpha value (reliability) did not reach optimal values, which led to the decision to eliminate items until the final structure was reached, in which factorial weights were obtained for all items, above .60 and represented by a single factor, thus, also achieving reliability values above .70 and an explained variance greater than 60% [29, 40–42].

Finally, a structure of four dimensions and 15 items was obtained [Relational Bond (N items = 4); Individualized Partnership (N items = 4); Professional Empowerment (N items = 3) and Therapeutic Communication (N items = 4)]. The final version of the PCTR-PT is included as S2 File (Spanish version) and S3 File (English version). The total variance explained was determined as 78.4%. The values of the factorial loads ranged from .642 to .897.

Until the best fit model was achieved, other alternatives were tested, which, as is usual in this type of study, did not show an adequate fit, and others that despite showing an acceptable fit were not the best. Model fit values of the hypothetical models tested as the result of CFA are as follows: 1) [(χ2 = 161,138; gl = 71, RMSEA = .059, GFI = .942, CFI = .975, TLI = .968, SMR = .034, and RMR = .024]; 2) [(χ2 = 176,240; gl = 83, RMSEA = .055, GFI = .941, CFI = .976, TLI = .969, SMR = .025, and RMR = .034]; 3) [(χ2 = 122,101; gl = 68, RMSEA = .047, GFI = .955, CFI = .985, TLI = .980, SMR = .028, and RMR = .024].

Table 2 shows the values of central tendency, variability, asymmetry, kurtosis and the percentage of minimum ("floor effect") and maximum ("ceiling effect") response for each of the items. The most outstanding feature is the asymmetry existing in the totality of the items, which was negative. A positive kurtosis was also observed in all items, with item 3 (My physiotherapist is kind towards me) standing out with a high kurtosis. All items showed a ceiling effect.

**Table 2:** Descriptive statistics of the items of the PCTR-PT scale.

	M	SD	skewness	kurtosis	% Floor	% Ceiling
1. I believe that my physiotherapist and I have connected.	4.64	.715	-3.133	13.866	.8	71.6
2. I feel that my physiotherapist provides me with the best possible care and attention.	4.71	.698	-3.762	18.317	1.1	78.1
3. My physiotherapist is kind towards me.	4.81	.564	-4.812	30.396	.8	85.2
4. I think that my physiotherapist is an accessible person.	4.73	.628	-3.605	18.085	.8	79.0
7. My physiotherapist is interested in how I am as a person and treats me individually.	4.29	1.022	-1.978	4.789	.5	54.4
8. My physiotherapist identifies my physical and/or emotional status and adjusts the treatment according to the same.	4.41	.904	-2.064	5.358	.8	59.6
11. My physiotherapist and I agree on what I want to achieve from the physiotherapy treatment.	4.40	.936	-2.189	5.731	1.9	59.3
12. My physiotherapist and I agree on which treatment to follow.	4.26	1.028	-1.702	3.010	2.2	53.3
17. When my physiotherapist explains exercises or health advice to me, he/she then asks about these and goes over them if necessary.	4.01	1.321	-1.352	.676	10.1	49.5
18. My physiotherapist makes me believe that I am able to get ahead with my own effort.	3.91	1.363	-1.246	.477	10.4	45.4
19. My physiotherapist makes me feel secure in what he says or does during the treatment process.	4.19	1.333	-1.680	1.477	10.9	61.2
27. The tone and volume of my physiotherapist’s voice generates trust.	4.53	.826	-2.631	8.601	2.5	65.3
28. My physiotherapist’s gaze generates trust.	4.48	.803	-2.404	8.337	1.4	60.4
29. I feel that my physiotherapist is interested in what I say.	4.54	.742	-2.488	9.211	1.4	62.6
30. My physiotherapist speaks to me in an easy and simple manner.	4.62	.726	-3.085	12.853	1.6	69.7

**PCTR-PT Scale**, The Person Centered Therapeutic Relationship in Physiotherapy Scale; **SD**, standard deviation.

**Fig 1:**
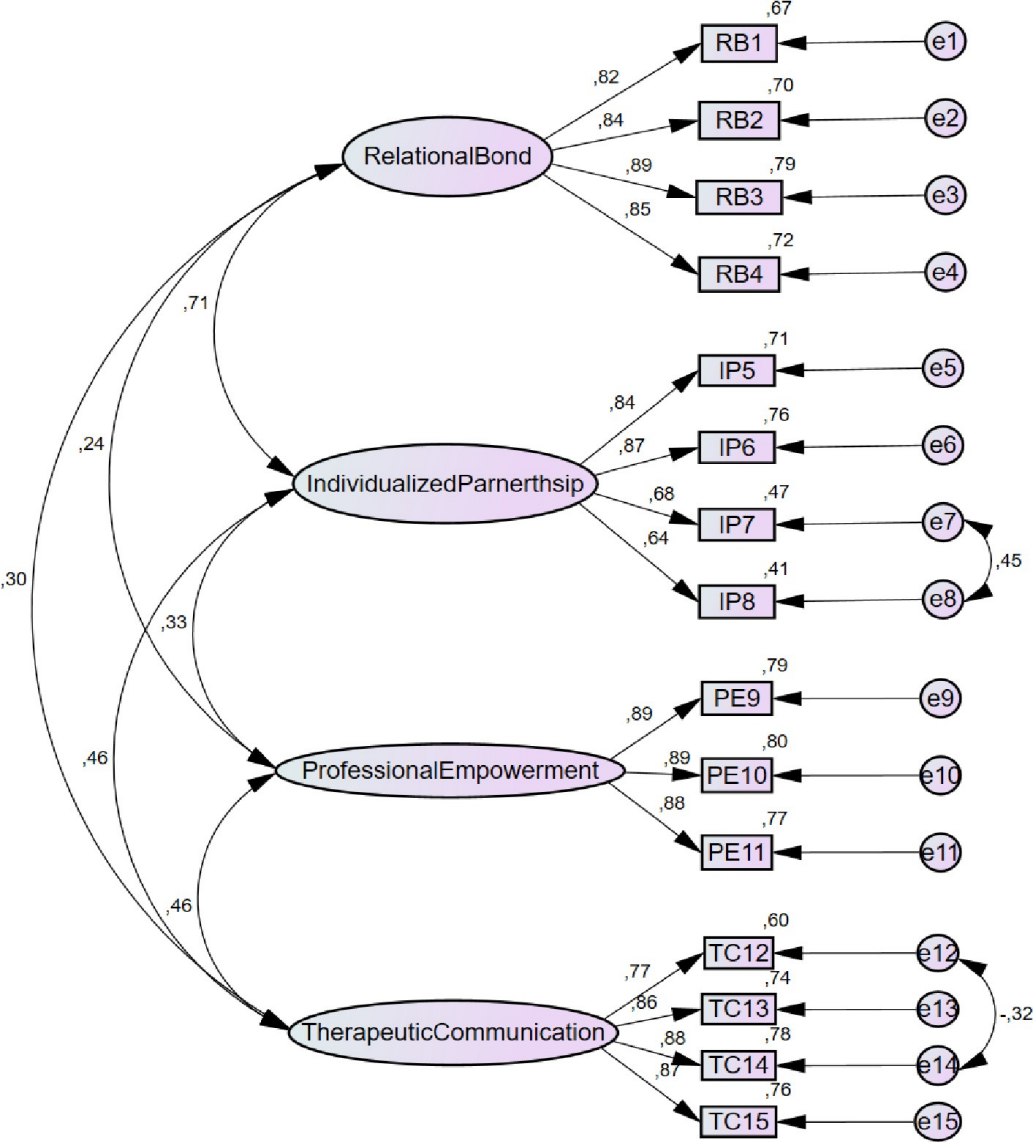
Confirmatory factor analysis of the four-dimensional model of the PCTR-PT.

Regarding the results of the confirmatory factor analysis, a four-factor model was constructed for which the standardized solution is shown in Fig 1 and the overall fit indices are shown in Table 3. The result of the chi-square test was significant (χ2(82) = 159,838; p < .0001), indicating that the hypothesis of a perfect model fit should be rejected. However, taking into account the problems associated with the use of this test, it was considered that other statistical tests were needed to evaluate the theoretical model in question. The remaining the indices analyzed are indicative of an acceptable model fit.

**Table 3:** Indices of goodness of fit of the confirmatory model.

INDEX	VALUE
CFI	.980
TLI	.974
GFI	.946
SRMR	.034
RMSEA	.051
RMR	024
Goodness of fit test	χ^2^ = 159.838; gl = 82; *P* < 0.0001
Reason for fit	χ^2^ / gl = 1,94 (<3)

**CFI**: Comparative Fit Index. **TLI:** Tucker-Lewis Index **GFI**: Goodness of Fit Index. **SRMR:** Standardized Root Mean Square Residual. **RMSEA**: Root Mean Standard Error of Approximation. **RMR**: Root Mean Residual.

Given that the study aim was to achieve the best possible fit for the scale, and despite the fact that many experts argue that there are never appropriate reasons to do so, on those occasions when the model's fit rates are considerably improved, covary error terms are a recommendable strategy. However, it should be used provided: it is applied within the same latent factor, there are strong theoretical arguments to support it [39], such as a possible overlap of content, similar item wording, reverse wording or with a differential tendency to social convenience, etc., the larger modification indices are initially addressed before addressing the smaller ones, the re-specification of the initial model can be justified [40, 42, 43].

Based on these arguments, the decision was made to correlate the errors e7 (item IP7) and e8 (item IP8) of the Individualized Partnership factor and the errors e12 (item TC12) and e14 (item TC14), belonging to the Therapeutic Communication factor, achieving a better model fit (Table 3) than that presented by the questionnaire without the application of this strategy [(χ2 = 243,371; gl = 84, RMSEA = .072, GFI = .918, CFI = .959, TLI = .948, SMR = .034, and RMR = .029].

Regarding the convergent validity analyzed by means of the Extracted Mean Variance (EMA) (Table 4), for the four dimensions, appropriate properties of convergent validity were reflected, since all of them met the EMA criterion > .50, with the Individualized Partnership dimension presenting the lowest value (.586). This means that each of the dimensions shares more than 50% of its variance with its items [42].

**Table 4:** Model validity measures of the PCTR-PT (composite reliability, CR; average variance extracted, AVE; maximum shared variance, MSV).

	CR	AVE	MSV	RB	IP	PE	TC	Reliability^a^	Convergent Validity^b^	Discriminant Validity^c^
RB	.912	.721	.504	**.849**				Ok	Ok	Ok
IP	.848	.586	.504	.710***	**.765**			Ok	Ok	Ok
PE	.915	.783	.209	.243***	.334***	**.885**		Ok	Ok	Ok
TC	.912	.722	.212	.296***	.460***	.457***	**.849**	Ok	Ok	Ok

RB = Relational Bond; IP = Individualized Partnership; PE = Professional Empowerment; TC = Therapeutic Communication; CR = Composite Reliability; AVE = Average Variance Extracted; MSV = Maximum Shared Variance.

† p < 0.100

* p < 0.050

** p < 0.010

*** p < 0.001.

a. CR >.70

b. CR > AVE, AVE > .50

c. MSV < AVE.

Regarding the discriminant validity analysis, the results showed that the MSV values were lower than the AVE for all four dimensions, in addition to the fact that the correlations between the constructs were lower than the square root of the AVE, thus confirming the presence of discriminant validity in statistical terms (Table 4).

The construct reliability was evaluated by calculating the Cronbach's alpha of the PCTR-PT scale (Table 5), observing that all values, both those of the scale (α = .884;) and those of each of the dimensions (α values between .86 and .91), were above .80

**Table 5:** Reliability statistics of the PCTR-PT (n = 366).

Scale/Dimension	Cronbach’s Alpha	N^o^ of items
PCTR-PT	.884	15
RB	.907	4
IP	.861	4
PE	.915	3
TC	.902	4

RB = Relational Bond; IP = Individualized Partnership; PE = Professional Empowerment; TC = Therapeutic Communication; CR = Composite Reliability.

For the analysis of the internal consistency of the scale, the composite reliability index (CR) was used, for which the results obtained were adequate for all four dimensions, as they were all above .70, varying between .84 (Individualized Partnership) and .91 (Professional Empowerment) (Table 4).

The analysis of the intraclass correlation coefficient (ICC) revealed excellent scores for both the total values (ICC = .900, F = 11.57, P<0.000) and the 95% confidence intervals (CI), which were between .84 and .94) for the measurement of PCTR-PT. For the scores of each of the dimensions, the values obtained ranged from .85 (Individualized Partnership) to .92 (Relational Bond) (Table 6).

**Table 6:** Internal consistency: Cronbach’s Alpha for each dimension and after item-reduction (n = 336), test-retest reliability comparing T1 with T2: ICC on scale level (n = 36) of the PCTR-PT.

Dimension	Cronbach’s Alpha T1	Cronbach’s Alpha T2	ICC	Confidence Interval ICC
PCTR-PT	.884	.914	.900	.846 - .941
RB	.907	.931	.928	.886 - .959
IP	.861	.852	.853	.768 - .916
PE	.915	.888	.875	.797 - .929
TC	.902	.909	.907	.853 - .947

RB = Relational Bond; IP = Individualized Partnership; PE = Professional Empowerment; TC = Therapeutic Communication; ICC = Intra-class correlation coefficient.

## Discussion

The purpose of the study was to determine the psychometric properties of the (PCTR-PT) with the aim of finding the most appropriate fit of the tool by identifying the number of dimensions and the total number of items that conform this tool.

The instrument was developed to assess the person-centered therapeutic relationship during the provision of physiotherapy services. The results obtained in this study show that the PCTR-PT scale has adequate psychometric properties in terms of internal consistency, temporal stability and construct validity. The CFA revealed an adequate fit of the structure with a total of 15 items distributed in four dimensions or factors.

The values obtained from the indices used to perform factorial validity and goodness-of-fit were acceptable, assuming a good fit of the model.

The results obtained for all the indices reflect a very good fit, as reflected in the values of the CFI, TLI, and GFI indices, which are either above .095 or very close to this value. The same applies to the error values (SRMR, RMR, RMSEA), which were equal to or less than .05 [40, 43–45] Particularly noteworthy are the values obtained for some indicators such as the IFC, TLI or RMSEA, which are some of the best references for showing the suitability of a model, since they do not depend on the size of the sample, which supports the viability of the measurement model [46]

With regard to the validity of the instrument, both convergent and divergent validity analyses presented adequate values for the indices used (AVE, MSV and correlations), thus confirming the suitability of the items to each of the dimensions to which they belong [35].

Regarding the internal consistency, both the values of the scale and of each of the dimensions showed adequate reliability values, considering that, in the development of a measurement tool, the minimum acceptable reliability is suggested to be equal or higher than 0.70 [47, 48] and a high coefficient would indicate the duplication or redundancy of some items [49, 50]. In our study all values were above .80, which is considered to reflect appropriate reliability of a construct, as the value is above 0.7) [33, 51]. The highest alpha value was found for the Professional Empowerment dimension. For the remaining dimensions (Individualized Partnership, Therapeutic Communication and Relational Bond) the alpha ranged from .861 to .907.

The composite reliability analysis was also adequate. According to the criterion proposed by Nunnally (1978) [47], the composite reliability should adopt a minimum value of .70 for modest reliability, with a higher value of .80 being more desirable for higher reliability. In our study all values were above .80 and ranged from .84 to .91.

Regarding the calculation of the reliability of the instrument, comparing T1 with T2, the ICC was excellent, presenting values above .90 for the whole scale and ranging from good to excellent (range of ICC .853 and .928) for each of the dimensions of the scale.

The PCTR-PT has some differential characteristics compared to other scales that measure the therapeutic relationship. The Working Alliance inventory (WAI) [19], the Helping Alliance Scale (HAS) [21] and the California Psychotherapy Alliance Scale (CALPAS) [22] exclusively measure the therapeutic alliance, a construct that is composed of bonding, agreement on the goals of treatment and agreement on tasks. The Scale to Assess the Therapeutic Relationship (STAR) [20] was designed to be used in the field of community mental health. The PCTR-PT, built within the scope of physical therapy and rehabilitation, focuses on dimensions or aspects related to the interaction between the physiotherapist and the person, deemed are necessary to carry out person-centered care (individualization [6, 7], empowerment [16, 52], agreement on the goals of treatment and treatment focused on people's preferences [16, 52, 53] and mutual trust [24]). This includes the communicative tools or necessary attitudes that the physiotherapist must have in order to establish a therapeutic relationship (verbal and non-verbal language that generates confidence [24, 28], a close attitude [54], sensitivity to change [55], active listening [15] or empathy [29]). Thus, the PCTR-PT not only values concepts needed to achieve person-centered care, but also professionals' interpersonal and communication skills.

One of the strengths of the PCTR-PT is the process of construction of the tool, in which 31 people with experience in receiving physiotherapy treatment participated. Thus, the generation of items was carried out based on their experiences and perceptions, collected via focus groups. We believe this is important to highlight this because in a recent review of tools for assessing competency in person-centered care [56], the authors concluded that "patients were not involved in the development of any of the assessment tools, which seems intrinsically paradoxical given the aims of PCC". In the same review, five scales measuring person-centered communication were analyzed, all designed to measure the doctor-patient relationship, three in primary care settings, one in radiation therapy settings, and one in settings for people with type 2 diabetes. To our knowledge, the PCTR-PT is the first scale to measure the person-centered therapeutic relationship in physical therapy settings.

Given the importance assigned to the therapeutic relationship as a necessary vehicle to carry out person-centered care and its necessary establishment in physiotherapy services, we believe that the PCTR-PT may be a valid tool for physiotherapists to understand their shortcomings in this area and to improve them. Moreover, a quality therapeutic relationship has been related to a greater adherence [57], treatment outcomes [23, 57] and improvements in patient satisfaction [58]. Therefore, health center managers could use it to improve the quality of care in physiotherapy centers.

Therefore, despite the need for further studies to confirm the factor structure of the PCTR-PT, it appears to be sound instrument, with a total of 15 items distributed across four dimensions or factors obtaining 15 items [Relational Bond (N items = 4); Individualized Partnership (N items = 4); Professional Empowerment (N items = 3) and Therapeutic Communication (N items = 4)], identified after exploratory analysis and confirmed by confirmatory factor analysis.

## Limitations

This study has several limitations that should be considered. First, all of the patients who participated in the study did so voluntarily and were selected consecutively by their physiotherapists, and therefore a selection bias may have occurred. Nonetheless, a large number of participants were recruited from different centers in Spain and their sociodemographic and clinical characteristics were very similar, therefore, these results can be generalized. Secondly, the test-retest reliability was carried out on a small sample, and although the results obtained were very good, it would be advisable to confirm these results with a larger sample. It would also be interesting to investigate the predictive capacity (sensitivity and specificity) of the PCTR-PT questionnaire.

## Conclusion

The results of our study show that the Person-Centered Therapeutic Relationship in Physiotherapy (PCTR-PT) Scale is a useful, valid and applicable instrument to assess the person-centered therapeutic relationship during physiotherapy interventions.

## Supporting information

S1 File (DOCX)

S2 File (DOCX)

S3 File (DOCX)

## References

[1] Sidani S, Fox M. Patient-centered care: clarification of its specific elements to facilitate interprofessional care. J Interprof Care 2014;28:134–41. 10.3109/13561820.2013.862519 24329714

[2] Wagner EH, Bennett SM, Austin BT, Greene SM, Schaefer JK, Vonkorff M. Finding Common Ground: Patient-Centeredness and Evidence-Based Chronic Illness Care. J Altern Complement Med 2005;11:s-7-s-15 10.1089/acm.2005.11.s-7 16332190

[3] Institute of Medicine. Shaping the Future; Crossing the quality chasm: a new health system for the 21th century. 2001 10.17226/10027.

[4] WHO. Integrated primary health care-based service delivery in the Global Conference on Primary Health Care, Astana, Kazakhstan. Astana: World Health Organization; 2018.

[5] Mead N, Bower P. Patient-centredness: a conceptual framework and review of the empirical literature. Soc Sci Med 2000;51:1087–110. 10.1016/s0277-9536(00)00098-8 11005395

[6] Morgan S, Yoder LH. A Concept Analysis of Person-Centered Care. J Holist Nurs 2012;30:6–15. 10.1177/0898010111412189 21772048

[7] Scholl I, Zill JM, Härter M, Dirmaier J. An integrative model of patient-centeredness—a systematic review and concept analysis. PLoS One 2014;9:e107828 10.1371/journal.pone.0107828 25229640 PMC4168256

[8] Santana MJ, Manalili K, Jolley RJ, Zelinsky S, Quan H, Lu M. How to practice person-centred care: A conceptual framework. Heal Expect 2018;21:429–40. 10.1111/hex.12640 29151269 PMC5867327

[9] Olsson LE, Jakobsson Ung E, Swedberg K, Ekman I. Efficacy of person-centred care as an intervention in controlled trials—a systematic review. J Clin Nurs 2013;22:456–65. 10.1111/jocn.12039 23231540

[10] Yun DW, Choi JS. Person-centered rehabilitation care and outcomes: A systematic literature review. Int J Nurs Stud 2019;93:74–83. 10.1016/j.ijnurstu.2019.02.012 30870614

[11] Byrne AL, Baldwin A, Harvey C. Whose centre is it anyway? Defining person-centred care in nursing: An integrative review. PLoS One 2020;15. 10.1371/journal.pone.0229923 32155182 PMC7064187

[12] Ree E, Wiig S, Manser T, Storm M. How is patient involvement measured in patient centeredness scales for health professionals? A systematic review of their measurement properties and content. BMC Health Serv Res 2019;19:12 10.1186/s12913-018-3798-y 30621682 PMC6323701

[13] Wijma AJ, Bletterman AN, Clark JR, Vervoort SCJM, Beetsma A, Keizer D, et al Patient-centeredness in physiotherapy: What does it entail? A systematic review of qualitative studies. Physiother Theory Pract 2017;33:825–40. 10.1080/09593985.2017.1357151 28820617

[14] Constand MK, Macdermid JC, Bello-haas VD, Law M. Scoping review of patient-centered care approaches in healthcare. BMC Health Serv Res 2014;14:1–9. 24947822 10.1186/1472-6963-14-271 PMC4079171

[15] Castro EM, Van Regenmortel T, Vanhaecht K, Sermeus W, Van Hecke A. Patient empowerment, patient participation and patient-centeredness in hospital care: A concept analysis based on a literature review. Patient Educ Couns 2016;99:1923–39. 10.1016/j.pec.2016.07.026. 27450481

[16] Zimmermann L, Konrad A, Müller C, Rundel M, Körner M. Patient perspectives of patient-centeredness in medical rehabilitation. Patient Educ Couns 2014;96:98–105. 10.1016/j.pec.2014.04.015 24862911

[17] Rathert C, Williams ES, Mccaughey D, Ishqaidef G. Patient perceptions of patient-centred care: Empirical test of a theoretical model. Heal Expect 2015;18:199–209. 10.1111/hex.12020 23176054 PMC5060773

[18] Bordin ES. The generalizability of the psychoanalytic concept of the working alliance. Psychother Theory, Res Pract 1979;16:252–60.

[19] Horvath AO, Greenberg LS. Development and validation of the Working Alliance Inventory. J Couns Psychol 1989;36:223–33.

[20] McGuire-Snieckus R, McCabe R, Catty J, Hansson L, Priebe S. A new scale to assess the therapeutic relationship in community mental health care: STAR. Psychol Med 2007;37:85–95. 10.1017/S0033291706009299 17094819

[21] Luborsky L, Barber JP, Siqueland L, Johnson S, Najavits LM, Frank A, et al The revised helping alliance questionnaire (HAq-II): Psychometric properties. J Psychother Pract Res 1996;5:260–71. 22700294 PMC3330423

[22] Barkham M, Agnew RM, Culverwell A. The California Psychotherapy Alliance Scales: A pilot study of dimensions and elements. Br J Med Psychol 1993;66:157–65. 10.1111/j.2044-8341.1993.tb01738.x 8353109

[23] Hall AM, Ferreira PH, Maher CG, Latimer J, Ferreira ML. The influence of the therapist-patient relationship on treatment outcome in physical rehabilitation: a systematic review. Phys Ther 2010;90:1099–110. 10.2522/ptj.20090245 20576715

[24] Pinto RZ, Ferreira ML, Oliveira VC, Franco MR, Adams R, Maher CG, et al Patient-centred communication is associated with positive therapeutic alliance: a systematic review. J Physiother 2012;58:77–87. 10.1016/S1836-9553(12)70087-5 22613237

[25] Morera-Balaguer J, Botella-Rico J, Martínez González M, Medina-Mirapeix F, Rodríguez Nogueira Ó. Physical therapists’ perceptions and experiences about barriers and facilitators of therapeutic patient-centred relationships during outpatient rehabilitation: a qualitative study. Brazilian J Phys Ther 2018;22:328–35. 10.1016/j.bjpt.2018.04.003 29705228 PMC6235755

[26] Miciak M, Mayan M, Brown C, Joyce AS, Gross DP. The necessary conditions of engagement for the therapeutic relationship in physiotherapy: an interpretive description study. Arch Physiother 2018;8:3 10.1186/s40945-018-0044-1 29468089 PMC5816533

[27] Miciak M, Mayan M, Brown C, Joyce AS, Gross DP. A framework for establishing connections in physiotherapy practice. Physiother Theory Pract 2019;35:40–56. 10.1080/09593985.2018.1434707 29432058

[28] O’Keeffe M, Cullinane P, Hurley J, Leahy I, Bunzli S, O’Sullivan PB, et al What Influences Patient-Therapist Interactions in Musculoskeletal Physical Therapy? Qualitative Systematic Review and Meta-Synthesis. Phys Ther 2016;96:609–22. 10.2522/ptj.20150240 26427530

[29] Morera-Balaguer J, Botella-Rico JM, Catalán-Matamoros D, Martínez-Segura R, Leal-Clavel M, Rodríguez-Nogueira Ó. Patients’ experience regarding therapeutic person-centered relationships in physiotherapy services: A qualitative study. Physiother Theory Pract 2019:1–11. 10.1080/09593985.2019.1603258 31002005

[30] Rodríguez Nogueira O, Botella-Rico J, Martínez González MC, Leal Clavel M, Morera-Balaguer J, Moreno-Poyato AR. Construction and content validation of a measurement tool to evaluate person-centered therapeutic relationships in physiotherapy services. PLoS One 2020;15:e0228916 10.1371/journal.pone.0228916 32119676 PMC7051061

[31] Díaz De Rada V. Estrategias para incrementar la tasa de respuesta en las encuestas. Rev Int Sociol 2018;59:133 10.3989/ris.2001.i29.759.

[32] Lorenzo-Seva U. Promin: A Method for Oblique Factor Rotation. Multivariate Behav Res 1999;34:347–65. 10.1207/S15327906MBR3403_3.

[33] Marôco J. Análise de equações estruturais: Fundamentos teóricos, software e aplicação. Pêro Pinheiro, Portugal: 2014.

[34] Hu L, Bentler PM. Cutoff criteria for fit indexes in covariance structure analysis: Conventional criteria versus new alternatives. Struct Equ Model A Multidiscip J 1999;6:1–55. 10.1080/10705519909540118.

[35] Hair JF, Babin B, Anderson R, Black W. Multivariate data analysis. 8th ed. Hampshire: 2018.

[36] Ramos A, Ramos A, Rosado A, Serpa S, Cangas A, Gallego J, et al Validity evidence of the Portuguese version of the Five Facet Mindfulness Questionnaire. Rev Psicol Del Deport 2018;27:87–98.

[37] Farrell AM. Insufficient discriminant validity: A comment on Bove, Pervan, Beatty, and Shiu (2009). J Bus Res 2010;63:324–7. 10.1016/J.JBUSRES.2009.05.003.

[38] Terwee CB, Bot SDM, de Boer MR, van der Windt DAWM, Knol DL, Dekker J, et al Quality criteria were proposed for measurement properties of health status questionnaires. J Clin Epidemiol 2007;60:34–42. 10.1016/j.jclinepi.2006.03.012 17161752

[39] Danny Xavier Arévalo Lozano CPP. Medición de la Confiabilidad del Aprendizaje del Programa RStudio Mediante Alfa de Cronbach. Rev Politécnica 2016;37:68–68.

[40] Bagozzi RP, Yi Y. On the evaluation of structural equation models. J Acad Mark Sci 1988;16:74–94. 10.1007/BF02723327.

[41] Fleiss JL. The design and analysis of clinical experiments. Hoboken, NJ, USA: Wiley; 2011.

[42] Fornell C, Larcker DF. Evaluating Structural Equation Models with Unobservable Variables and Measurement Error. J Mark Res 1981;18:39 10.2307/3151312.

[43] Brown TA. Confirmatory factor analysis for applied research. Second Edi. New York: The Guildford Press; 2015 10.1680/geot.8.B.012.

[44] Byrne BM. Structural Equation Modeling With AMOS. Basic Concepts, Applications, and Programming 3rd ed. New York: Routledge 2016

[45] Kline RB. Principles and practice of structural equation modeling Third Ed. New York: Guilford 2011.

[46] Hu L, Bentler PM. Fit indices in covariance structure modeling: Sensitivity to underparameterized model misspecification. Psychol Methods 1998;3:424–53. 10.1037/1082-989X.3.4.424.

[47] Nunnally JC, Bernstein IH. Psychometric theory. 3rd ed. New York:McGraw-Hill 1994.

[48] Bland JM, Altman DG. Cronbach’s alpha. BMJ 1997;314:572 10.1136/bmj.314.7080.572 9055718 PMC2126061

[49] Jaju A., & Crask MR. The perfect design: Optimization between reliability, validity, redundancy in scale items and response rates. Am Mark Assoc 1999;10:127–31.

[50] DeVellis RF. Scale development: theory and applications. Fourth ed. Los Angeles:Sage Publishing 2016

[51] Kline RB. Principles and practice of structural equation modeling. Third ed. New York: Guilford publications 2015.

[52] Nolte E, Merkur S, Anell A. The person at the centre of health systems: an introduction Achiev. Pers. Heal. Syst., Cambridge: Cambridge University Press; 2020, p. 1–18. 10.1017/9781108855464.004.

[53] Håkansson Eklund J, Holmström IK, Kumlin T, Kaminsky E, Skoglund K, Höglander J, et al “Same same or different?” A review of reviews of person-centered and patient-centered care. Patient Educ Couns 2019;102:3–11. 10.1016/j.pec.2018.08.029 30201221

[54] Del Baño-Aledo ME, Medina-Mirapeix F, Escolar-Reina P, Montilla-Herrador J, Collins SM. Relevant patient perceptions and experiences for evaluating quality of interaction with physiotherapists during outpatient rehabilitation: a qualitative study. Physiotherapy 2014;100:73–9. 10.1016/j.physio.2013.05.001. 23778264

[55] Medina-Mirapeix F, Jimeno-Serrano FJ, Escolar-Reina P, Del Baño-Aledo ME. Is patient satisfaction and perceived service quality with musculoskeletal rehabilitation determined by patient experiences? Clin Rehabil 2013;27:555–64. 10.1177/0269215512468142 23258933

[56] Ekman N, Taft C, Moons P, Mäkitalo Å, Boström E, Fors A. A state-of-the-art review of direct observation tools for assessing competency in person-centred care. Int J Nurs Stud 2020;109:103634 10.1016/j.ijnurstu.2020.103634 32531569

[57] Kelley JM, Kraft-Todd G, Schapira L, Kossowsky J, Riess H. The influence of the patient-clinician relationship on healthcare outcomes: A systematic review and meta-analysis of randomized controlled trials. PLoS One 2014;9:e94207 10.1371/journal.pone.0094207 24718585 PMC3981763

[58] Medina-Mirapeix F, Oliveira-Sousa SL, Sobral-Ferreira M, Montilla-Herrador J, Jimeno-Serrano FJ, Escolar-Reina P. What elements of the informational, management, and relational continuity are associated with patient satisfaction with rehabilitation care and global rating change? Arch Phys Med Rehabil 2013;94:2248–54. 10.1016/j.apmr.2013.04.018 23643715

