# Supplementary material for: The psychometric properties of the person-centered therapeutic relationship in physiotherapy scale
Source: PLoS One. 2020 Nov 6;15(11):e0241010. doi: 10.1371/journal.pone.0241010 (PMC7647106; doi:10.1371/journal.pone.0241010)
Supplement: S2 File — (DOCX) [file pone.0241010.s002.docx]

CUESTIONARIO DE RELACIÓN TERAPÉUTICA CENTRADA EN LA PERSONA EN FISIOTERAPIA

Este cuestionario está dirigido a CONOCER LA RELACIÓN TERAPÉUTICA ENTRE LOS PACIENTES Y SUS FISIOTERAPEUTAS

Diversos estudios han demostrado su importancia en los procesos de recuperación del paciente

SU información nos puede AYUDAR a MEJORAR LA RELACIÓN TERAPÉUTICA

INSTRUCCIONES para rellenar el cuestionario:

## 1) A continuación aparece una lista de afirmaciones y preguntas acerca de sus experiencias personales con su fisioterapeuta. Piense y señale qué categoría de respuesta describe mejor su propia experiencia.

## 2) Lea bien las preguntas y las respuestas

## 3) NO DEJE preguntas SIN contestar.

3) *Señale así* ⌧ *las respuestas adecuadas* (hágalo con cuidado para no equivocarse).

**Indique su grado de acuerdo con respecto a las siguientes afirmaciones:**

*1. Creo que mi fisioterapeuta y yo hemos conectado.*

| Totalmente de acuerdo |  | De acuerdo |  | Ni de acuerdo ni en desacuerdo |  | En desacuerdo |  | Totalmente en  desacuerdo |
| --- | --- | --- | --- | --- | --- | --- | --- | --- |

*2. Siento que mi fisioterapeuta me proporciona el mejor cuidado y atención posibles.*

| Totalmente de acuerdo |  | De acuerdo |  | Ni de acuerdo ni en desacuerdo |  | En desacuerdo |  | Totalmente en  desacuerdo |
| --- | --- | --- | --- | --- | --- | --- | --- | --- |

*3. Mi fisioterapeuta es amable conmigo.*

| Totalmente de acuerdo |  | De acuerdo |  | Ni de acuerdo ni en desacuerdo |  | En desacuerdo |  | Totalmente en  desacuerdo |
| --- | --- | --- | --- | --- | --- | --- | --- | --- |

*4. Creo que mi fisioterapeuta es una persona accesible.*

| Totalmente de acuerdo |  | De acuerdo |  | Ni de acuerdo ni en desacuerdo |  | En desacuerdo |  | Totalmente en  desacuerdo |
| --- | --- | --- | --- | --- | --- | --- | --- | --- |

*5. Mi fisioterapeuta se interesa en cómo soy como persona y me trata de manera individual*

| Totalmente de acuerdo |  | De acuerdo |  | Ni de acuerdo ni en desacuerdo |  | En desacuerdo |  | Totalmente en  desacuerdo |
| --- | --- | --- | --- | --- | --- | --- | --- | --- |

*6. Mi fisioterapeuta identifica mi estado físico y/o emocional y ajusta el tratamiento en función del mismo*

| Totalmente de acuerdo |  | De acuerdo |  | Ni de acuerdo ni en desacuerdo |  | En desacuerdo |  | Totalmente en  desacuerdo |
| --- | --- | --- | --- | --- | --- | --- | --- | --- |

*7. Entre mi fisioterapeuta y yo nos ponemos de acuerdo sobre lo que yo quiero conseguir con el tratamiento de Fisioterapia.*

| Totalmente de acuerdo |  | De acuerdo |  | Ni de acuerdo ni en desacuerdo |  | En desacuerdo |  | Totalmente en  desacuerdo |
| --- | --- | --- | --- | --- | --- | --- | --- | --- |

*8. Mi fisioterapeuta y yo nos ponemos de acuerdo sobre el tratamiento a seguir*

| Totalmente de acuerdo |  | De acuerdo |  | Ni de acuerdo ni en desacuerdo |  | En desacuerdo |  | Totalmente en  desacuerdo |
| --- | --- | --- | --- | --- | --- | --- | --- | --- |

*9. Cuando mi fisioterapeuta me explica ejercicios o consejos para mi salud, después me pregunta por ellos y los revisa si es necesario.*

| Totalmente en desacuerdo |  | En desacuerdo |  | Ni de acuerdo ni en desacuerdo |  | De  acuerdo |  | Totalmente de acuerdo |
| --- | --- | --- | --- | --- | --- | --- | --- | --- |

*10. Mi fisioterapeuta me hace creer que tengo la capacidad para salir adelante con mi propio esfuerzo.*

| Totalmente en desacuerdo |  | En desacuerdo |  | Ni de acuerdo ni en desacuerdo |  | De  acuerdo |  | Totalmente de acuerdo |
| --- | --- | --- | --- | --- | --- | --- | --- | --- |

*11. Mi fisioterapeuta me transmite seguridad en lo que dice o hace en el proceso de tratamiento.*

| Totalmente en desacuerdo |  | En desacuerdo |  | Ni de acuerdo ni en desacuerdo |  | De  acuerdo |  | Totalmente de acuerdo |
| --- | --- | --- | --- | --- | --- | --- | --- | --- |

*12. El tono y el volumen de voz de mi fisioterapeuta me generan confianza.*

| Totalmente en desacuerdo |  | En desacuerdo |  | Ni de acuerdo ni en desacuerdo |  | De  acuerdo |  | Totalmente de acuerdo |
| --- | --- | --- | --- | --- | --- | --- | --- | --- |

*13. La mirada de mi fisioterapeuta me genera confianza.*

| Totalmente en desacuerdo |  | En desacuerdo |  | Ni de acuerdo ni en desacuerdo |  | De  acuerdo |  | Totalmente de acuerdo |
| --- | --- | --- | --- | --- | --- | --- | --- | --- |

14. *Siento que mi fisioterapeuta se interesa por lo que le digo.*

| Totalmente en desacuerdo |  | En desacuerdo |  | Ni de acuerdo ni en desacuerdo |  | De  acuerdo |  | Totalmente de acuerdo |
| --- | --- | --- | --- | --- | --- | --- | --- | --- |

15. *Mi fisioterapeuta me habla de manera fácil y sencilla.*

| Totalmente en desacuerdo |  | En desacuerdo |  | Ni de acuerdo ni en desacuerdo |  | De  acuerdo |  | Totalmente de acuerdo |
| --- | --- | --- | --- | --- | --- | --- | --- | --- |

**¡MUCHAS GRACIAS POR SU COLABORACIÓN!**
